# Mesocarnivores affect hispid cotton rat (*Sigmodon hispidus*) body mass

**DOI:** 10.1038/s41598-019-51168-y

**Published:** 2019-10-10

**Authors:** Gail Morris, L. Mike Conner

**Affiliations:** Jones Center at Ichauway, Newton, Georgia 39870 USA

**Keywords:** Behavioural ecology, Community ecology, Population dynamics

## Abstract

Predator communities are changing worldwide: large carnivores are declining while mesocarnivores (medium-sized mammalian predators) are increasing in number and ecological influence. Predator choice of prey is not random and different predators select prey with different characteristics. Changes in predator communities can change predation patterns experienced by prey. Little is known about how mesocarnivore communities influence prey morphology. We used 14 years of mark-recapture data to investigate how mesocarnivore exclusion affected body mass of hispid cotton rats (*Sigmodon hispidus*). Finding adult male cotton rats were 9% heavier with mesocarnivore exclusion, we developed hypotheses to explain this observation. Greater adult male body mass in exclosures resulted from: (1) a non-significant trend of increased survival of large males, (2) faster juvenile male growth during the fall and a similar non-significant trend among adult males, and (3) spatial partitioning by size among males. Taxa-specific predation rates (i.e., rates of predation by snakes, raptors, or mesocarnivores) did not differ among male body mass classes. Mesocarnivores disproportionately preyed on large females while raptors targeted small females, but female body mass was not influenced by mesocarnivore exclusion. Changes in predator communities can result in multiple small effects that collectively result in large differences in prey morphology.

## Introduction

Predation occurs when a predator kills its prey. The simplicity of this definition belies the complexity of the predation process. Although the most obvious result of predation is that prey abundance is reduced by the number of animals killed, choice of prey by the predator, and prey responses to risk of predation, can greatly influence how predators affect prey populations. Risk of predation varies across the lifespan of many species, often being particularly great during periods of vulnerability such as when individuals are very young or when caring for young^1,2^. In other species, older prey may escape predation by growing to a size at which their gape-limited predators are incapable of predation^3^. Not all age classes contribute equally to population growth; therefore, loss of prey in age classes which contribute little to population growth may have relatively little effect on prey population dynamics, whereas loss of individuals with the potential to contribute more to the population is likely to have a larger impact^4,5^.

Prey are often at risk from multiple predators, and different predators may focus on different life stages or sizes of their prey. For example, in a lab experiment with hispid cotton rats (*Sigmodon hispidus*, hereafter cotton rats), Roberts and Wolfe^6^ found dominant cotton rats were disproportionally preyed on by feral cats, while hawks preferred subordinate individuals. Dominant cotton rats tend to be more active^6^ and larger^7,8^. Roberts and Wolfe^6^ suggested the difference in activity rates between dominant and subordinate individuals, and differences in mode of hunting between raptors and mammalian predators (the cats preyed on the first individual which moved while the hawks did not appear to select prey based on activity) explained their results. Because life stages vary in their contributions to population growth and different predators may select prey with different characteristics, shifts in predator communities may result in shifts in prey population dynamics. Shifts in predator communities may also influence evolutionary responses of prey^9,10^.

Prey with multiple predators with variable hunting modes face a dilemma whereby behavioural responses to limit risk from one predator may increase risk of predation from another. For example, gerbils (*Gerbillus* spp) in the Negev Desert face predation from snakes and owls. Owls present the greatest risk in open habitat while snakes are generally encountered near shrubs^11^. Shifts in predator communities may therefore change how prey respond to predation risk. Behavioural responses to predation risk may result in indirect effects (e.g., reductions in activity, shifts in habitat use) which can be as large or larger than direct effects (i.e., lethal) of predation^12^; therefore, shifts in predator communities will likely also lead to shifts in the indirect effects of predation experienced by prey populations.

Predator communities are changing worldwide; apex predator populations are declining^13^, often resulting in increased mesocarnivore populations^14–17^ (here we define mesocarnivores as mammalian predators with a body mass <34 kg and an average of 13–16 km)^18^. Although effects of mesocarnivores on prey abundance have been well studied^19–23^, there has been little investigation into how changes in predator communities influence other aspects of prey such as body mass and growth. Recent work suggests apex predators and human harvest may influence the morphology of prey^9,24^ but effects of mesocarnivores on prey morphology have been little studied. Because of the worldwide pervasiveness of shifts in predator communities toward increased mesocarnivore populations, and because hunting modes vary among predator species, such effects may be common.

Here, we examine mechanisms by which mesocarnivores influence their prey; specifically, how exclusion of mesocarnivores affected body mass of hispid cotton rats. We focused on cotton rats because they serve as important prey for a variety of predators on our study area (i.e., mesocarnivores, snakes, and raptors)^2,25,26^ and in the southern United States in general^27–30^, they are the most common prey species on our study site, and because we have long-term mark-recapture and radio-telemetry data available for this species. We focused on body mass because perceived predation risk commonly influences body size and growth^9,31–34^ and because, among rodents, population growth rate is strongly influenced by age of sexual maturity and fertility^35^, both of which are related to body mass^36,37^, making this parameter potentially significant in terms of population dynamics. Upon finding that adult male cotton rats were significantly larger in exclosures than in controls, we developed multiple hypotheses to evaluate mechanisms for this observation^38^ using mark-recapture and radio-telemetry data. Specifically, we examined growth rates, body mass of juveniles at first capture, and size-specific capture probability, survival, and mortality among cotton rats.

## Methods

### Study site

This research was carried out at the Jones Center at Ichauway in Baker County, Georgia, USA. The Jones Center is an 11,700 ha property comprised primarily of longleaf pine (*Pinus palustris*) and wiregrass (*Aristida stricta*) savannah. Slash pine (*Pinus elliotti*), loblolly pine (*Pinus taeda*), mixed pine and hardwood forests, and hardwood bottoms also occur on the site. Most of the property is managed using prescribed fire on a 2-year rotation which maintains a forest structure with a diverse groundcover, a relatively open over-story, and limited mid-story.

Cotton rat predators in this region include mesocarnivores, raptors, and snakes^25^. Mesocarnivores include coyotes (*Canis latrans*), foxes (*Vulpes vulpes* and *Urocyon cinereoargenteus*), raccoons (*Procyon lotor*), Virginia opossums (*Didelphis virginiana*), nine-banded armadillos (*Dasypus novemcinctus*), striped skunks (*Mephitis mephitis*), and bobcats (*Lynx rufus*), although opossums, armadillos, and skunks are unlikely to present much risk to cotton rats^26,39–42^. The snake community in this region is diverse. The snake species most likely to prey on cotton rats include gray rat snakes (*Pantherophis spiloides*), corn snakes (*Pantherophis guttatus*), Eastern diamondback rattlesnakes (*Crotalus adamanteus*), timber rattlesnakes (*Crotalus horridus*), and coachwhips (*Masticophis flagellum*)^25,29^. Snake activity is seasonal, thus snake predations in winter are rare^25^. Raptor activity also varies seasonally. Permanent residents likely to prey on cotton rats include red-tailed hawks (*Buteo jamaicensis*), red-shouldered hawks (*Buteo lineatus*), barn owls (*Tyto alba*), eastern screech-owls (*Megascops asio*), great horned owls (*Bubo virginianus*), and barred owls (*Strix varia*)^27,43–45^. During the winter, northern harriers (*Circus hudsonius*) are also a threat.

### Field methods

In 2003, we built 4 approximately 40 ha mesocarnivore exclosures. Exclosure plots were surrounded by a 0.9 m tall woven wire (5 × 10 cm mesh) fence with electrified lines at the top, middle, and bottom. Small mammals, raptors, and snakes had unfettered access to exclosures but fencing and electric lines discouraged mesocarnivore entry. Exclosures were monitored seasonally using track count surveys^46^ and camera trapping to detect mesocarnivore trespass. Mesocarnivores were removed by trapping if detected. Each exclosure plot was paired with a nearby control in which predators had unrestricted access.

Each exclosure and control plot contained a small mammal trapping grid. Grids were 12 × 12 with 15 m spacing between stations. We used Sherman live traps (model XLK, H.B. Sherman Traps, Tallahassee, FL, USA) baited with oats and bird seed. We trapped from 2003 through early 2017. From 2005 through 2017, granular bifenthrin insecticide was sprinkled around each trap to prevent red-imported fire ant (*Solenopsis invicta*) predation on captured small mammals. Trapping was conducted in each plot once seasonally except: no trapping occurred during the summer and fall of 2004; from summer 2007 through spring 2009, 2 trapping sessions occurred each season; from fall 2009 through winter 2011, all 8 sites were trapped during summer, but only 1 control and 1 exclosure were trapped during other seasons. Traps were set for 4 nights and checked once each morning. Traps were closed during daytime when the weather was hot (>27 °C) and polyester fiber and extra food were provided during cold weather (<5 °C). We did not trap during full moons^47^. For each captured animal, we recorded location, species, sex, body mass, age (adult or juvenile, based on mass), reproductive condition (for females, lactating and/or pregnant or neither, for males, with testes descended or not), and hind foot length. Body mass was measured using a Pesola spring scale (Baar, Switzerland) with a 300 g capacity and 2 g gradations (accuracy ± 0.3%). Trapping and handling followed recommendations of the Animal Care and Use Committee of the American Society of Mammalogists^48^. Our methods were approved by the Jones Center at Ichauway under the Georgia Department of Natural Resources scientific collecting permit number 1000528068.

Prescribed fires occurred in all sites during the winters of odd years (i.e., 2003, 2005, 2007, etc.). Supplemental feeding occurred in 2 exclosure and 2 control grids from the summer of 2007 through the summer of 2009^49–51^.

From the summer of 2006 through the summer of 2009, cotton rats weighing ≥90 g were radio-collared^25,49,50^. Status of collared rats as alive or dead was verified visually at least once a week. When rats were found dead we attempted to identify the source of mortality using evidence at the site^25^.

### Statistical methods

#### Assessing mass and developing hypotheses

From the trapping dataset, we selected capture records for cotton rats and separated adults (≥50 g)^52^ from juveniles. We further separated males and females for all analyses because mass changes influenced by pregnancy and lactation may obscure body mass effects caused by the predator exclusion treatment, which was our primary interest. We excluded all captures from controls and exclosures which were supplementally fed from the summer of 2007 to the summer of 2009 as part of a separate research project^49–51^. Additionally, one of the control grids was adjacent to a field which was periodically supplementally fed as part of northern bobwhite (*Colinus virginianus*) management activities. Because rodents commonly consume such food^53^, we removed all records of individuals captured at stations on the half of the trapping grid nearest this field. Because repeated captures may cause changes in small mammal body mass^54,55^, we used only the first capture record for each individual in a trapping session.

We assessed the effect of the predator exclusion treatment, season, and their interaction on body mass of adult cotton rats using a linear model implemented in the MIXED procedure in SAS version 9.4 (SAS Institute Inc., Cary, NC, 2012). We included season because activity of some cotton rat predators vary seasonally in our region (i.e., snakes and raptors). Here and in subsequent analyses, we assessed importance of site (i.e., trapping grid) as a random effect by first fitting the model with the random effect but no fixed effects, using the restricted maximum likelihood method. If the covariance parameter estimate for the random effect was zero or near zero (i.e., > orders of magnitude smaller than the residual variance), we did not include it in the model which included the fixed effects of interest. Otherwise, site was retained as a random effect^56^. We set α = 0.05 (two-tailed) for all statistical tests. In all analyses, normality assumptions were checked by examining normal probability plots and plots of residuals vs predictions. Given a significant outcome, post-hoc multiple comparisons were carried out to determine which levels among factors differed significantly. We used the Bonferroni correction to correct for alpha-inflation associated with post-hoc tests^57^.

**Figure 1:**
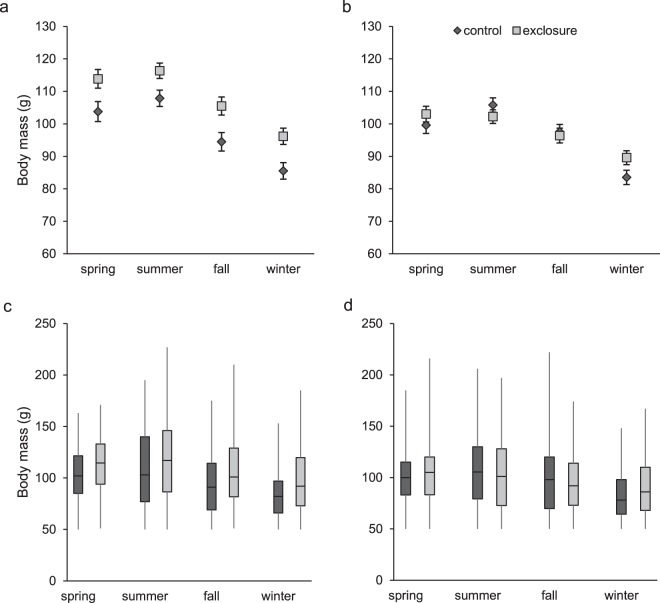
Least square mean body mass (±) for adult male (**a**) and adult female (**b**) hispid cotton rats (*Sigmodon hispidus*) in plots where mesocarnivores were excluded (exclosure) and where predators had unrestricted access (control). Box plots for males (**c**) and females (**d**) show the distribution of the raw body mass data. Data were collected as part of a mark-recapture study from 2003–2017 in Baker County, Georgia, USA.

**Figure 2:**
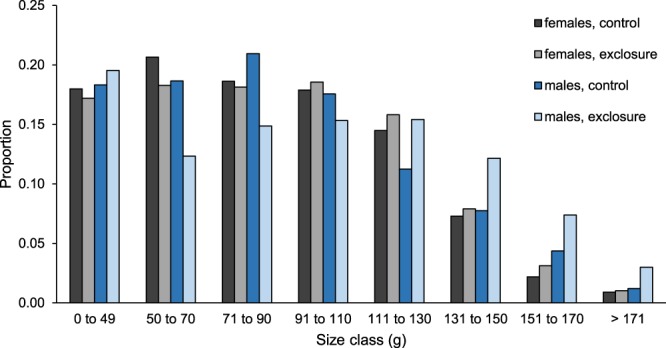
Proportion of juvenile (<50 g) and adult (≥50 g) hispid cotton rat (*Sigmodon hispidus*) captures in each of seven size classes in plots where mesocarnivores were excluded (exclosure) and where predators had unrestricted access (control). Data were collected as part of a mark-recapture study from 2003–2017 in Baker County, Georgia, USA.

Adult male, but not female, cotton rats were significantly larger in mesocarnivore exclosures than in controls (Figs 1 and 2; see Results for details). To determine the mechanism responsible for this difference, we developed a set of hypotheses which we then evaluated using long-term mark-recapture and radio-telemetry data. These hypotheses included: (1) small and non-significant differences in survival and/or growth rates between exclosures and controls accumulated over time such that they led to large differences in body mass after fourteen years of study, (2) size-specific differences in capture probability between exclosures and controls gave the appearance of larger body mass in exclosures when no such difference actually existed, (3) juveniles and/or adults had faster growth rates in exclosures than in controls, (4) juveniles were larger at birth in exclosures than controls, (5) mesocarnivores preferentially preyed on large cotton rats^6^, and exclusion of mesocarnivores caused large cotton rats to have better survival and be more abundant in exclosures than in controls, and (6) increased abundance of larger and more dominant males forced smaller subordinates into suboptimal habitat^7^ where smaller males experienced greater mortality, compounding the effect hypothesized in 5. Previous analyses of mark-recapture and known-fate radio telemetry data in these plots^25,50^ indicated nuanced differences in cotton rat survival relative to predator exclusion; however, these analyses did not consider body size. Therefore, our analysis for hypothesis 5 focused on effects of size on survival.

Individuals in populations exposed to high rates of predation may experience strong selective pressure on rates of maturation and reproduction, leading to increases in these rates over several generations (i.,e., a possible mechanism for hypothesis 1)^58–60^. Predators may also influence individual growth rates of prey (as in hypothesis 3) positively (by removing competition) or negatively (by intimidating prey into reducing foraging)^32,33^. Given that previous investigations in these study sites found no strong effect of mesocarnivore exclusion on cotton rat abundance^50^, we expect differences in cotton rat density unlikely to have influenced any differences in growth rates between controls and exclosures.

Predation risk is also known to influence body mass of prey offspring, usually negatively^31,59^. Although we did not have records of body mass of cotton rat offspring at birth, hypothesis 4 was tested using body mass of juveniles at first capture.

Size-specific predation has been observed in many predator-prey interactions, most prominently in aquatic systems^58–60^, and may have profound effects on prey populations. Hypothesis 5 was tested in two ways: (1) we used cause-specific mortality (i.e., we estimated rates of predation by taxa including mesocarnivores, snakes, and raptors) and body mass data associated with radio-collared cotton rats to determine whether size-specific predation occurred among cotton rats, and (2) we estimated survival of radio-collared cotton rats of different size classes in controls and exclosures.

Hypothesis 6 was tested by analyzing rates at which cotton rats of different sizes were captured at the same trapping station in a given session. Although this hypothesis does not relate directly to predation risk, we included it to investigate whether social interactions among cotton rats may influence individual risk of predation.

Collectively, these hypotheses include mechanisms by which direct (e.g., size specific predation) and indirect (e.g., individual growth rates) effects of predation may influence body mass. These hypotheses are not mutually exclusive and multiple mechanisms may simultaneously influence prey body mass.

Although the impetus for conducting these analyses was to understand the mechanism by which male rats came to be larger in exclosures than in controls, we also analyzed data associated with females. Sex-specific differences in how traits such as maturation and growth respond to predation have commonly been observed^31,61^. The different reproductive demands males and females face may influence the decisions each makes in response to predation risk. We analyzed data associated with both male and female cotton rats to gain a fuller understanding of how the mesocarnivore community affects their prey and why predator effects may differ between males and females.

#### Adult body mass effects over time

At the time of this analysis, our study sites had been monitored for 14 years. We hypothesized that differences in predator communities may have contributed to natural selection resulting in gradually increasing body mass in the absence of mesocarnivores. Using the mark-recapture dataset described above, we used a linear model implemented in the MIXED procedure in SAS to assess the effects of the predator exclusion treatment, year, and their interaction on body mass of adult males and females. We included site as a random effect.

#### Adult growth rate

We assessed growth rates of adult male and female cotton rats in controls and exclosures by identifying all individuals from the body mass analysis which were captured in >1 trapping session. We calculated daily growth rate by subtracting the first body mass from the second and dividing by the number of days between captures. We assessed whether growth rate was influenced by body mass on initial capture, season, the predator exclusion treatment, and the interaction of predator exclusion and season using a linear model implemented in the MIXED procedure in SAS, treating site as a random effect. Some individuals were captured on more than two occasions. For these, we calculated a separate growth rate over each interval between captures.

Because the model assessing treatment effects on growth rate is linear and growth rates are often not linear (e.g., younger animals typically grow faster^62^), we further explored predator exclusion effects on growth rate by dividing adults into 4 size classes, based on initial body mass. The size classes were: 50 to 80 g, 81 to 110 g, 111 to 140 g, and >140 g. We assessed the effects of the predator exclusion treatment, initial size class, and their interaction on rate of mass change using a linear model implemented in the MIXED procedure in SAS, using site as a random effect.

#### Juvenile body mass on first capture and growth rate

As with adults, we assessed the effects of predator exclusion, season, and the interaction of predator exclusion and season on body mass of male and female juveniles, with site included as a random effect. We then identified all juveniles which were recaptured in a subsequent trapping session and calculated daily growth rate (g/day). We did not include juveniles for which the interval between captures spanned more than one season. Male and female juvenile growth rates were compared between controls and exclosures using a linear model implemented in the MIXED procedure in SAS which predicted juvenile growth rate as a consequence of mesocarnivore exclusion, season, the interaction between treatment and season, and number of months between captures. Site was not included as a random effect, according to the criteria described previously. For males, sample sizes for winter and spring were small (N = 4 during springs, all in exclosures; N = 3 during winters, with only 1 from an exclosure), so the analysis used only juvenile males first captured in summer and fall.

#### Size-specific survival and cause-specific predation

To determine effects of body mass and mesocarnivore exclusion on cotton rat survival, we used known-fate telemetry data to estimate survival over a period of 30 days from body mass measurement. We used data from cotton rats which were radio-collared from the summer of 2006 through the summer of 2009^25,50^. We separated collared rats into 2 size classes: 90 to 110 g and ≥130 g. We did not collar rats weighing <90 g due to minimum size requirements to carry radio collars. We discarded data associated with rats in the 111 to 129 g range to limit the chance that rats near a size-class boundary would gain or lose enough weight to be in a different size class at the time of death or at the end of the 30 days. Rats with unknown fates (i.e., experienced transmitter failure or went missing) or which were judged to have died from causes other than predation (e.g., died in a trap, ran over by a tractor) during the 30 day period were censored. We then assessed whether survival of cotton rats through 30 days was influenced by the additive and interactive effects of the predator exclusion treatment and size class using a Cox proportional hazards regression^63^ implemented in the PHREG procedure in SAS.

We additionally examined the possibility that different taxa of predators disproportionally preyed on different size classes of cotton rats using cause-specific mortality data from radio-collared cotton rats. Using the known-fate data described above, we identified rats judged to have been killed by a predator within 30 days of body mass being recorded. We determined the number of rats killed by each predator type (avian, mammalian, snake, and unknown) in each size class and calculated the proportion of rats which would be expected to be preyed upon if each predator type preyed among size classes at random. For each predator type, we used Fisher’s exact tests to determine if large and small cotton rats were preyed upon as expected.

#### Size-specific capture probability

To determine whether the difference in adult male body mass between mesocarnivore exclosures and controls could have been caused by mass-specific differences in capture probabilities (e.g., if large rats had greater capture probability in exclosures than in controls) rather than real differences in body mass, we used a Huggins closed-capture model^64^ to estimate capture probability as a function of the interaction between body size and predator exclusion. We divided male cotton rats into 4 size classes for this analysis: 0 to 50 g, 51 to 90 g, 91 to 110 g, and >110 g. These classes were chosen because they represented the classes in which differences (or similarities) between controls and exclosures were most distinct (see Fig. 2) and therefore would be most useful in determining whether the observed differences in body mass could have resulted from biased capture rates. The model was implemented in Program MARK^65^ using the Program R 3.5.0^66^ package RMark 2.2.4^67^.

#### Competitive interactions among male cotton rats

We investigated competitive interactions among male cotton rats by examining size-specific capture patterns from the trapping dataset. We identified all occasions in which 2 different males were caught at the same station during a given trapping session, excluding occasions in which a female was captured between 2 males. For the same reasons associated with estimating capture probabilities described above, we classified individuals into 4 size classes: 0 to 50 g, 51 to 90 g, 91 to 110 g, and >110 g. We counted the number of times male captures of each size were followed by a male of the same or different size. We analyzed these data using a 4 × 4 Chi-square test using the FREQ procedure in SAS. Given a significant result, we conducted pairwise comparisons using 2 × 2 Chi-square tests for each size combination to determine which capture patterns differed from expectation.

## Results

### Efficacy of mesocarnivore exclusion

Between July of 2004 and March of 2017, we detected 378 mesocarnivores in controls (171 raccoon, 59 bobcat, 48 armadillo, 36 fox, 24 opossum, 18 coyote, 7 unidentified canid, and 15 unknown mesocarnivores) and 89 in exclosures (30 raccoon, 6 bobcat, 37 armadillo, 2 fox, 7 opossum, 1 coyote, 1 skunk, and 5 unknown mesocarnivores). The coyote trespass occurred when flooding caused a week-long power outage at 1 exclosure. Importantly, armadillos do not prey on small mammals and were not removed when exclosures were originally established. If armadillo counts are excluded, mesocarnivore use of controls was approximately 6.3 × greater than that of exclosures, indicating substantial reduction in mesocarnivore use of exclosures.

### Adult body mass

We examined data associated with 1610 captures of 1310 adult male cotton rats. Predator exclusion and season did not interact (*F* = 0.14; *d.f*._*n,d*_ = 3, 1596; *P* = 0.94, Fig. 1a) to affect body mass. Adult males were larger (*F* = 15.12; *d.f*._*n.d*._ = 1, 1596; *P* < 0.01) in exclosures (108.0 ± 1.8 g; least square mean ± SE; *N* = 861) than controls (97.9 ± 1.8 g, *N* = 749). Small (<110 g) adult males were more prevalent in controls, while large males were more common in exclosures (Fig. 2). Season also influenced (*F* = 40.76; *d.f*._*n.d*._ = 3, 1596; *P* < 0.01) body mass (Fig. 1a).

We examined data associated with 2155 captures of 1482 adult female cotton rats. Unlike males, there was a significant interactive effect of season and predator exclusion on body mass (Fig. 1b, *F* = 3.28; *d.f*._*n.d*._ = 3, 2141; *P* = 0.02). We made 4 post-hoc comparisons to examine the interaction, resulting in an adjusted α = 0.003 and found no differences in adult female body mass between exclosures and controls in any season (all cases *P* ≥ 0.05; Fig. 1b).

### Adult body mass over time

**Figure 3:**
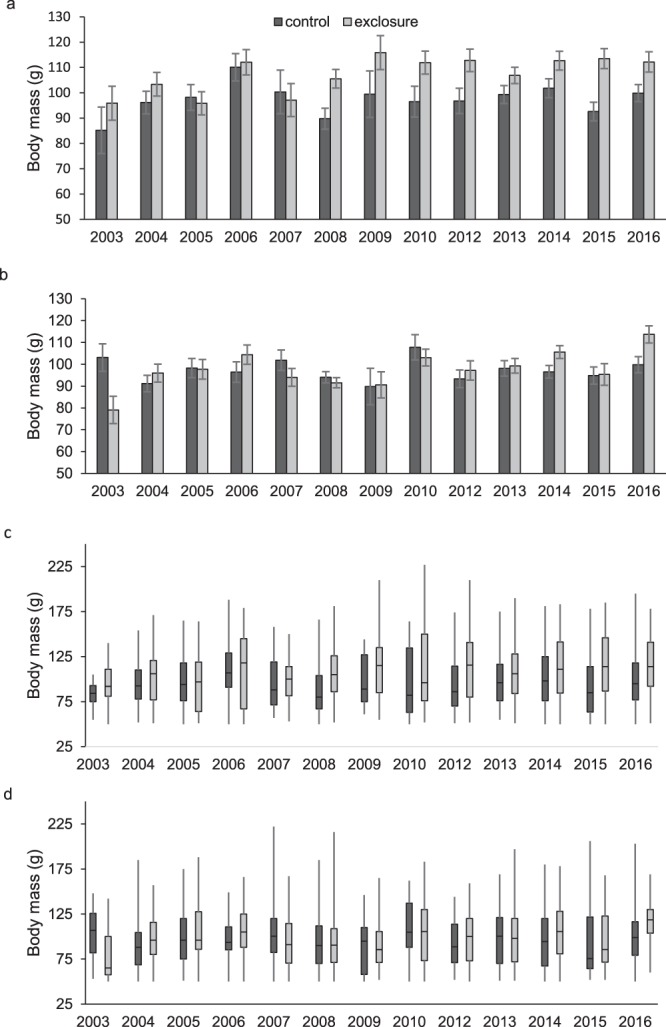
Least square mean adult male (**a**) and female (**b**) hispid cotton rat (*Sigmodon hispidus*) body mass (±SE) in plots where mesocarnivores were excluded (exclosure) and where predators had unrestricted access (control) by year. Box plots for males (**c**) and females (**d**) show distributions of raw data. Data for 2011 are not shown because no adult males were captured in controls. Each “year” includes data from spring, summer, and fall of that year, plus winter of the following year (e.g., 2012 includes spring, summer, and fall of 2012 and winter of 2013). Means were calculated this way because prescribed fires were carried out between winter and spring sessions, in February or March of odd years. Data were collected as part of a mark-recapture study from 2003–2017 in Baker County, Georgia, USA.

In 11 of 13 years, adult male cotton rats were larger in exclosures than controls (Fig. 3a,c). Data from 2011 were not included due to low capture rates (only 6 in exclosures and 0 in controls). There was no interaction between predator exclusion and year (*F* = 1.26; *d.f*._*n.d*._ = 12, 1572; *P* = 0.23), but both predator exclusion and year influenced body mass (*F* = 10.64; *d.f*._*n.d*._ = 1, 1572; *P* < 0.01 and *F* = 2.15; *d.f*._*n.d*._ = 12, 1572; *P* = 0.01, respectively). Among adult females, the interaction between year and predator exclusion significantly influenced body mass (*F* = 2.37; *d.f*._*n.d*._ = 12, 2116; *P* < 0.01). We made 13 post-hoc comparisons to examine this interaction and adjusted α to 0.004 to account for alpha inflation. However, there was no difference in adult female body mass between controls and exclosures in any year (*P* > 0.004, Fig. 3b,d).

### Adult growth rates

**Figure 4:**
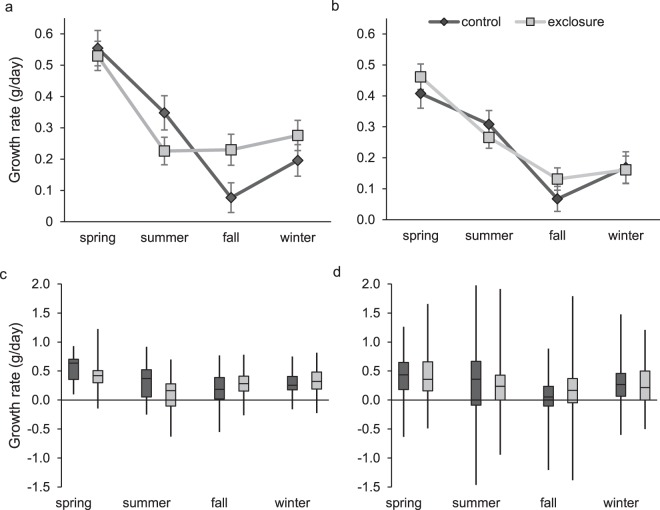
Least square mean seasonal growth rates (g/day, ±SE) of adult male (**a**) and female (**b**) hispid cotton rats (*Sigmodon hispidus*) in plots where mesocarnivores were excluded (exclosure) and where predator access was unrestricted (control). Least square mean estimates were generated from models which included additive and interactive effects of season and the predator exclusion treatment and an additive effect of body mass on initial capture. Box plots for males (**c**) and females (**d**) show distributions of raw data. Data were collected as part of a mark-recapture study from 2003–2017 in Baker County, Georgia, USA.

We used 303 adult male (120 in controls and 183 in exclosures) and 677 adult female (275 in controls and 402 in exclosures) cotton rats to calculate growth rates. Male growth rate was influenced by an interaction between the predator exclusion treatment and season (*F* = 5.07; *d.f*._*n,d*._ = 3, 288; *P* = 0.002; Fig. 4a,c). Within season pairwise comparisons between exclosures and controls were not significant after adjusting α for multiple comparisons (α = 0.013); however, fall growth rates of males in exclosures (0.23 ± 0.05 g/day) compared to controls (0.08 ± 0.05 g/day) approached significance (*t* = −2.24, *d.f*. = 288, *P* = 0.026). Adult male body mass on initial capture influenced growth rate (*F* = 134.88; *d.f*._*n,d*_ = 1, 288; *P* < 0.001), as did season (*F* = 36.64; *d.f*._*n,d*_ = 3, 288; *P* < 0.001). Among females, there was no interaction between predator exclusion and season (*F* = 0.90; *d.f*._*n,d*._ = 3, 662; *P* = 0.44; Fig. 4b,d) and predator exclusion did not influence growth rates (controls: 0.24 ± 0.03 g/day; exclosures, 0.26 ± 0.02 g/day; *F* = 0.24; *d.f*._*n,d*_ = 1, 662; *P* = 0.62). However, body mass at first capture and season both influenced female growth (*F* = 284.20; *d.f*._*n,d*_ = 1, 662; *P* < 0.001 and *F* = 27.45; *d.f*._*n,d*_ = 3, 662; *P* < 0.001, respectively).

**Figure 5:**
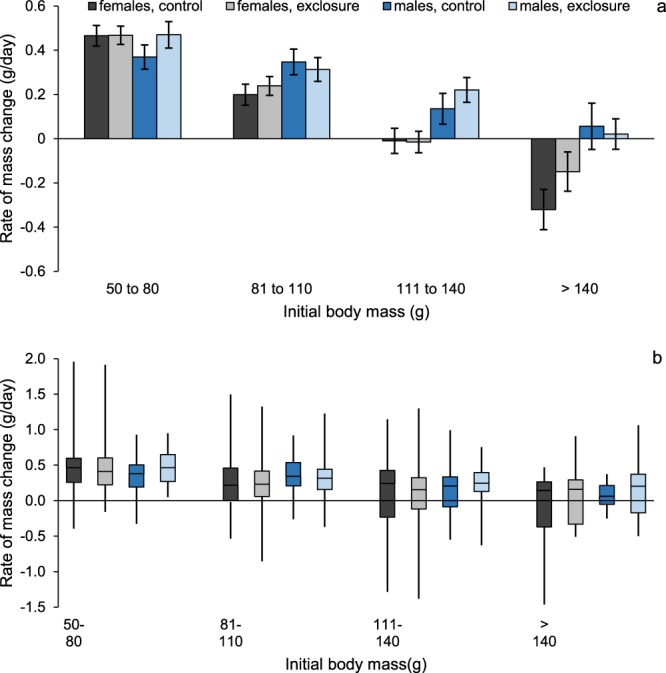
Least square mean (**a**) size-specific growth rates (g/day, ±SE) of adult hispid cotton rats (*Sigmodon hispidus*) in plots where mesocarnivores were excluded (exclosure) and where predator access was unrestricted (control) by initial size class. Box plots (**b**) show distributions of raw data. Data were collected as part of a mark-recapture study from 2003–2017 in Baker County, Georgia, USA.

Examination of size-class specific growth rates indicated no interaction between predator exclusion and size class for either sex (males: *F* = 1.42; *d.f*._*n,d*_ = 3, 289; *P* = 0.24, and females: *F* = 0.66; *d.f*._*n,d*_ = 1, 663; *P* = 0.58) and no effect of predator exclusion (males: *F* = 0.17; *d.f*._*n,d*_ = 1, 289; *P* = 0.68, and females: *F* = 0.92; *d.f*._*n,d*_ = 1, 663; *P* = 0.34). Size on first capture was a predictor of growth rate for both males (*F* = 17.74; *d.f*._*n,d*_ = 3, 289; *P* < 0.001) and females (*F* = 69.15; *d.f*._*n,d*_ = 3, 663; *P* < 0.001). As expected, smaller individuals grew faster (Fig. 5).

### Juvenile body mass on first capture and growth rate

**Figure 6:**
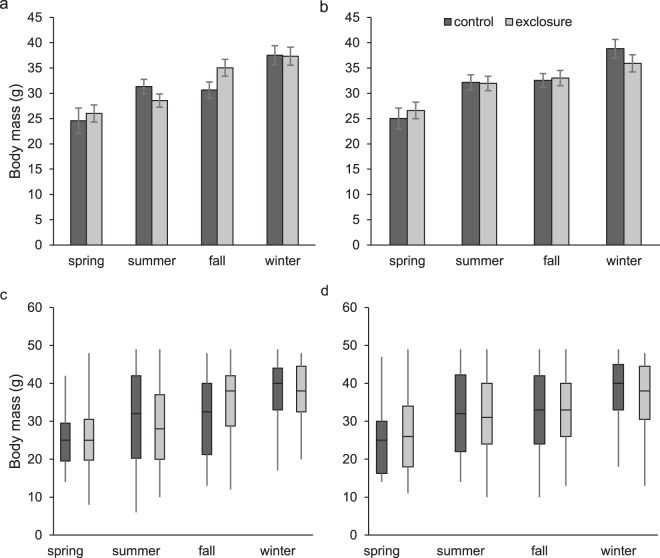
Seasonal least square mean juvenile male (**a**) and female (**b**) hispid cotton rat (*Sigmodon hispidus*) body mass (±SE) in plots where mesocarnivores were excluded (exclosure) and where predator access was unrestricted (control). Box plots for males (**c**) and females (**d**) show distributions of raw data. Data were collected as part of a mark-recapture study from 2003–2017 in Baker County, Georgia, USA.

Records of 377 males (168 in controls and 209 in exclosures) and 459 females (222 in controls and 237 in exclosures) were used for the analyses of juvenile body mass. There was no interaction between the predator exclusion treatment and season for either sex (males: *F* = 2.36; *d.f*._*n,d*_ = 3, 363; *P* = 0.07, and females: *F* = 0.75; *d.f*._*n,d*_ = 3, 445; *P = *0.53). For both sexes, body masses were similar (males: *F* = 0.23; *d.f*._*n,d*_ = 1, 363; *P* = 0.63; and females: *F* = 0.03; *d.f*._*n,d*_ = 1, 445; *P = *0.86) in controls (males: 31.0 ± 1.1 g, females: 32.1 ± 1.1 g) and exclosures (males: 31.7 ± 1.0 g; females: 31.9 ± 1.1 g). Season influenced both juvenile male (*F* = 15.58; *d.f*._*n,d*_ = 3, 363; *P* < 0.001; Fig. 6) and juvenile female body mass (*F* = 17.68; *d.f*._*n,d*_ = 3, 445; *P* < 0.001; Fig. 6).

**Figure 7:**
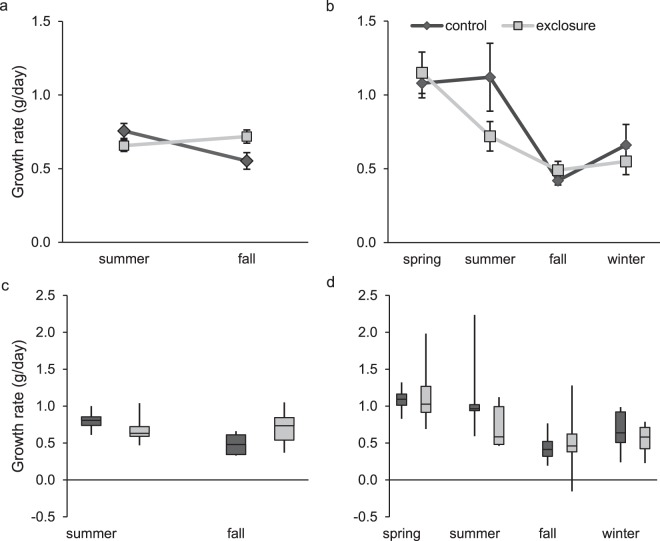
Least square mean male (**a**) and female (**b**) juvenile hispid cotton rat (*Sigmodon hispidus*) growth rates (±SE) by season in plots where mesocarnivores were excluded (exclosure) and where predator access was unrestricted (control). Box plots for males (**c**) and females (**d**) show distributions of raw data. Spring and winter growth rates are not shown for males due to low sample sizes. Data were collected as part of a mark-recapture study from 2003–2017 in Baker County, Georgia, USA.

Thirty-eight juvenile males from the preceding analysis were recaptured in a subsequent trapping session (15 in controls and 23 in exclosures). Predator exclusion and season interacted (Fig. 7; *F* = 7.69; *d.f*._*n,d*_ = 1, 33; *P* = 0.01) to influence juvenile male growth rates. We conducted 2 post-hoc comparisons to examine the interaction with an adjusted of α = 0.025. During fall, juvenile males grew faster in exclosures (0.72 ± 0.04 g/day) than in controls (0.55 ± 0.06 g/day; *t* = − 2.34, *d.f*. = 33, *P* = 0.025). Summer growth rates were similar in controls and exclosures (*t* = 1.58, *d.f*. = 33, *P* = 0.12). Juvenile male growth had an inverse relationship with months between captures (i.e., longer intervals between captures resulted in smaller growth rates; *F* = 13.46, *d.f*._*n,d*_ = 1, 33; *P* = 0.001).

We calculated growth rates for 86 juvenile females (42 in controls and 44 in exclosures). Predator exclusion and season did not interact to affect juvenile female growth (Fig. 7; *F* = 2.49; *d.f*._*n,d*_ = 3, 77; *P* = 0.07) and growth rate did not differ (*F* = 1.76; *d.f*._*n,d*_ = 1, 77; *P* = 0.19) between controls (0.81 ± 0.06 g/day) and exclosures (0.71 ± 0.05 g/day). Growth rate was not affected by months between captures (*F* = 0.37; *d.f*._*n,d*_ = 1, 77; *P* = 0.55). However, juvenile female growth varied by season (Fig. 7; *F* = 10.49; *d.f*._*n,d*_ = 3, 77; *P* < 0.001).

### Size-specific survival and cause-specific predation

**Figure 8:**
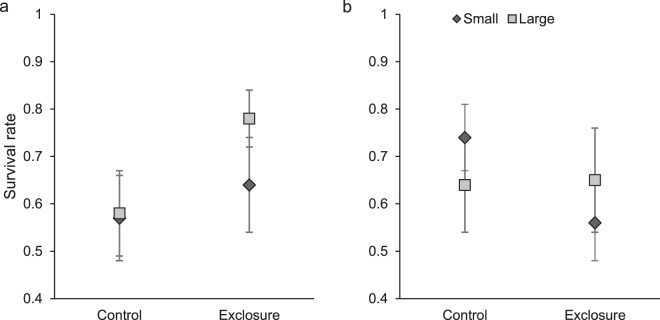
Survival of small (90 to 110 g) and large (≥130 g) male (**a**; N small control = 37, N large control = 33, N small exclosure = 22, N large exclosure = 46) and female (**b**; N small control = 46, N large control = 27, N small exclosure = 47, N large exclosure = 18) radio-collared hispid cotton rats (*Sigmodon hispidus*) through 30 days (±SE) following body mass measurement in plots where mesocarnivores were excluded (exclosure) and where predator access was unrestricted (control). Data were collected from 2006–2009 in Baker County, GA, USA.

We used 138 male and 138 female radio-monitored cotton rats to examine size-specific survival over 30 days (Fig. 8). Among males, the interaction between size class and predator exclusion (*Χ*^2^ = 0.83; *d.f*. = 1; *P* = 0.36), size class (*Χ*^2^ = 0.002; *d.f*. = 1; *P* = 0.97), and predator exclusion (*Χ*^2^ = 0.23, *d.f*. = 1, *P* = 0.63) were not predictors of survival. However hazard ratios were larger for males in controls than exclosures, and this was especially true for large males, although this difference was not significant at α = 0.05 (Table 1).

**Table 1:** Hazard ratios with 95% lower and upper confidence levels (LCL and UCL, respectively) and *P*-values associated with Cox proportional hazards regressions assessing the effects of mesocarnivore exclusion, body size, and their interaction on survival within 30 days of body mass measurement of radio-collared hispid cotton rats (*Sigmodon hispidus*).

		Hazard ratio	Hazard ratio LCL	Hazard ratio UCL	*P*
Males	In exclosures, large vs small	0.56	0.22	1.43	0.23
	In controls, large vs small	0.98	0.46	2.09	0.97
	Among large, control vs exclosure	2.16	0.94	4.92	0.07
	Among small, control vs exclosure	1.24	0.52	2.95	0.65
Females	In exclosures, large vs small	0.74	0.30	1.85	0.53
	In controls, large vs small	1.48	0.61	3.57	0.39
	Among large, control vs exclosure	1.05	0.37	2.95	0.93
	Among small, control vs exclosure	0.53	0.25	1.10	0.09

Data were collected from 2006–2009 in Baker County, Georgia, USA. Small rats weighed 90–110 g and large rats were ≥130 g.

Among females, the interaction between size class and predator exclusion (*Χ*^2^ = 1.14, *d.f*. = 1; *P* = 0.29), predator exclusion (*Χ*^2^ = 2.89; *d.f*. = 1; *P = *0.09), and size class (*Χ*^2^ = 0.75; *d.f*. = 1; *P* = 0.39) did not influence survival (Table 1).

We used mortalities of 52 male and 55 female cotton rats attributed to predation to evaluate size-specific patterns of predation. Among males, there were no differences (*P* > 0.05) between observed and expected rates of predation by size class for any predator type (Table 2). Among females, mammalian predators were more likely (*Χ*^2^ = 5.46, *d.f*. = 1, *P* = 0.03) to prey on large rather than small rats (Table 2). The opposite was true of avian predators (*Χ*^2^ = 4.99, *d.f*. = 1, *P* = 0.04; Table 2).

**Table 2:** Cause-specific mortality of male and female hispid cotton rats (*Sigmodon hispidus*) radio-collared in southwest Georgia, USA, from 2006–2009.

Body mass class	N avian predations	N expected avian predations	N snake predations	N expected snake predations	N mammalian predations	N expected mammalian predations	N unknown predations	N expected unknown predations
**Males**								
Small	8	8.0	3	4.0	6	6.0	9	8.0
Large	8	8.0	5	4.0	6	6.0	7	8.0
*P*		1.00		0.70		1.00		0.77
**Females**								
Small	20	16.1	6	7.4	2	4.7	9	8.7
Large	4	7.9	5	3.6	5	2.3	4	4.3
*P*		0.04		0.47		0.03		1.00

Number of deaths attributed to each predator type is given, as is the number that would be expected if each predator type preyed among size classes at random. Small rats weighed 90–110 g and large rats were >130 g. Outcomes of Fisher’s exact tests comparing observed and expected mortality rates among size classes are given at the bottom of the table for each predator type.

### Size-specific capture probability

For all size classes, male cotton rat capture probabilities were greater in exclosures than in controls but only significantly greater for juveniles (Table 3). Therefore, biased capture rates did not influence our observation of larger adult male cotton rats in mesocarnivore exclosures.

**Table 3:** Capture probabilities for male hispid cotton rats (*Sigmodon hispidus*) by size class in plots which excluded mesocarnivores (exclosures) and in control plots where mesocarnivores had unrestricted access.

Size class (g)	Control		Exclosure	
	Estimate (±SE)	95% CI	Estimate (±SE)	95% CI
0 to 50	0.30 (±0.02)	0.26–0.34	0.42 (±0.02)	0.38–0.46
51 to 90	0.38 (±0.02)	0.35–0.41	0.41 (±0.02)	0.38–0.44
91 to 110	0.38 (±0.02)	0.34–0.43	0.42 (±0.02)	0.38–0.47
>110	0.42 (±0.02)	0.38–0.46	0.45 (±0.01)	0.42–0.47

Capture probabilities were estimated using a Huggins closed-capture model. Standard errors (SE) and 95% confidence intervals (CI) are also given. Data were collected from 2003–2017 in Baker County, GA, USA.

### Competitive interactions among male cotton rats

On 741 occasions, 2 different male cotton rats were trapped at the same trap station during a given trapping session in which no females were trapped between male captures. Size class of subsequent captures was dependent on size class of the first capture (*Χ*^2^ = 59.87, *d.f*. = 9, *P* < 0.0001; Table 4). Four pairwise comparisons were significant after adjusting α for multiple comparisons (α = 0.003; Table 4). Within a given trapping session, juvenile males (0 to 50 g) were more likely to be trapped after a juvenile male. Similarly, males weighing 51 to 90 g were more likely to be trapped after a male of similar size, as were males weighing >110 g. For the size class 91 to 110 g, there was a non-significant (after Bonferroni correction) trend following the same pattern (*P* = 0.004). Additionally, males weighing >110 g were less likely to be trapped following a male weighing 51 to 90 g.

**Table 4:** Number of times multiple male hispid cotton rats (*Sigmodon hispidus*) were captured at the same trap station during a given trapping session.

1^st^ capture size class	2^nd^ capture size class	No. times observed	*Χ* ^2^	*d.f*.	*P*
0–50 g	0–50 g	44	28.33	1	<0.001
0–50 g	51–90 g	41	0.80	1	0.371
0–50 g	91–110 g	12	7.33	1	0.007
0–50 g	>110 g	35	1.54	1	0.215
51–90 g	0–50 g	35	1.97	1	0.160
51–90 g	51–90 g	107	16.27	1	<0.001
51–90 g	91–110 g	42	0.03	1	0.857
51–90 g	>110 g	56	9.85	1	0.002
91–110 g	0–50 g	17	2.41	1	0.120
91–110 g	51–90 g	42	0.58	1	0.448
91–110 g	91–110 g	34	8.10	1	0.004
91–110 g	>110 g	40	0.07	1	0.791
>110 g	0–50 g	33	2.83	1	0.093
>110 g	51–90 g	65	7.24	1	0.007
>110 g	91–110 g	39	0.09	1	0.762
>110 g	>110 g	99	19.26	1	<0.001

Observations are given by size class. A 4 × 4 Chi-square test of these data was significant (*P* < 0.001, *d.f*. = 9, *Χ*^2^ = 59.87). Pairwise comparisons were then conducted for each pair of size classes. *P*-values (*P*), degrees of freedom (*d.f*.), and Chi-square values (*Χ*^2^) for each test are shown. To correct for α inflation associated with post-hoc tests, comparisons were considered significant at α = 0.003 (Bonferroni correction).

## Discussion

Predator communities are in flux worldwide due to the loss of apex predators, release of mesocarnivore populations, introduction of exotic species, and removal of predators associated with the management of livestock, game, and species of conservation concern^13,68–72^. Further, predators affect prey populations in complex ways that can be influenced by a predator’s hunting mode, preference for prey with certain physical characteristics, or preference for hunting in certain habitats^3,6,73^. Changes in predator communities may therefore cause shifts in both direct (lethal) and indirect (sublethal) effects of predators on their prey. Lethal effects of predators on prey have received a great deal of research attention^74,75^ and sublethal effects of predators on prey are becoming increasingly apparent^12,76–80^.

Investigations regarding predator influence on prey morphology are few, especially in terrestrial ecosystems, but evidence suggests such effects occur^9,81,82^ and, in some cases, in ways which could influence prey population dynamics. For example, the loss of raptors from wind farms in India led to cascading effects on fan-throated lizard (*Sarada superba*) populations including increases in lizard abundance, declines in lizard body condition, and duller dewlap coloration^81^. Among ungulates, risk of predation from large predators may drive selection for large body size and defensive morphology (e.g., horns^9^). In some cases, invasive predators have had trans-generational effects on prey morphology and survival, as with eastern fence lizards (*Sceloporus undulatus*) from areas invaded by red-imported fire ants (*Solenopsis invicta*) in the southeastern United States^82^. Here we used results from a long-term mesocarnivore exclusion experiment to investigate how the mesocarnivore guild influences morphology of a common prey species in the southern United States, the hispid cotton rat.

Body mass of adult males was 9% greater in mesocarnivore exclosures than in controls where mesocarnivores had unrestricted access. We examined multiple hypotheses which could, individually or in combination, explain the mechanism responsible for this. There was no evidence that size-specific capture probabilities could have given the appearance of larger males in exclosures in the absence of a real difference. Further, the greater adult male body mass in exclosures does not appear to have accrued over time as the difference in mass has been apparent since the earliest years of the study (i.e., there was no evidence of selection for more rapid growth over time). Juvenile male body mass was similar between controls and exclosures, and we found no evidence of size-specific patterns of predation among radio-collared males. However, large (>130 g) radio-collared males had better survival through 30 days in exclosures than controls, although this trend was not statistically significant. Additionally, during the fall, juvenile males grew faster in exclosures than in controls. Adult males demonstrated a similar, but non-significant, trend. Therefore, our data suggest the greater adult male body mass observed in mesocarnivore exclosures resulted from a combination of faster growth (in some seasons) combined with better survival of large males in predator exclosures. The reduced survival of small, relative to large, males in exclosures was likely influenced by competitive interactions between males which further contributed to the observed difference in mass. These results indicate cotton rats experience both direct and indirect effects of predation which collectively influence body mass. Social interactions likely compound these effects.

Our trapping records suggest that males segregate spatially by body size. Within trapping sessions, captures of males of a given size class were positively and significantly associated with future captures of males of the same size class (with the exception of the 91–110 g size class which approached significance). For juveniles, this may be attributed to capture of multiple individuals from the same litter. However, it is unlikely that relatedness could explain these patterns among the larger size classes given the high mortality rates of cotton rats and competitive interactions of adults^62,83^. We suggest these size-specific capture patterns support observations that dominant cotton rats monopolize preferred habitat while subordinate rats are found in marginal habitats^7^. Monopolization of preferred habitats by large rats contributes to our explanation of the size difference between controls and exclosures. We suggest small rats in both exclosures and controls are forced into marginal habitat. However, the presence of mesocarnivores reduces the advantage of being large within controls, while in exclosures the greater abundance of large rats may make small rats especially likely to be excluded from good habitat. Conversely, greater predation rates on large rats in controls may open better habitat to smaller rats, allowing smaller rats to remain more abundant within controls than exclosures.

Predation risk can affect growth rates by influencing food intake and energy expenditures of prey^32,61,84^. Predation can also influence growth rates by thinning populations and reducing competition for resources^32,81^. Mesocarnivore exclusion resulted in faster growth rates, especially among juveniles, but only among males during the fall season. Juveniles of both sexes are closely associated with their mothers, use similar habitats, and presumably have similar diets^83^. Factors influencing juvenile cotton rat growth include the mother’s size^85^ and diet^37,86^, litter size^87^, season^88^, weather^89^, and age at weaning^90^. However, cotton rats mature rapidly and although all individuals included in the juvenile growth rate analyses were juveniles on the first body mass measurement, 92 to 94% (males and females, respectively) were of adult body mass on the second capture. Therefore, some juvenile growth occurred in rats that had become independent of their mother. Young males in controls may have perceived greater predation risk than young males in exclosures and made behavioural changes resulting in slower growth rates. Growth rates of young females may not have been similarly influenced by mesocarnivore exclusion because females have different energetic demands associated with achieving breeding condition, or because females respond to predation risk differently than males. Tidhar *et al*.^61^ found that exposure to predator scent affected body mass of male and female bank voles (*Clethrionomys glareolus*) differently. They hypothesized that this occurred because delaying breeding (which is strongly associated with body mass in rodents) may be more costly for females than for males. Therefore, males may be more likely to reduce foraging and food intake than females in response to predation risk. Male cotton rats in our control plots may have responded similarly to male bank voles, behaving in ways that led to reduced growth rates while females accepted higher risk to avoid delays in reproductive maturity.

Doonan and Slade^91^ observed that food supplementation increased proportion of juveniles and small adult cotton rats. Thus, the differences in body mass we observed could have been caused by better food availability in controls leading to a younger and smaller structured population. Cherry *et al*.^92^ observed a trophic cascade in our study plots resulting from a preference of white-tailed deer (*Odocoileus virginianus*) for mesocarnivore exclosures and a greater rate of browsing in exclosures. However, we captured proportionally more juvenile male cotton rats in exclosures than in controls. Further, the difference in cotton rat body mass was observed in the first year of the study, before cascading effects were manifest. Thus, it is unlikely that food availability explains differences in male cotton rat mass.

Among rodents, including cotton rats^91^, large males are more likely to be reproductive and have larger testes, which are associated with greater breeding success^93,94^. Therefore, even in the absence of lethal effects of predation, there is potential for population-level consequences to result from mesocarnivore exclusion. However, previous research on our study plots indicated no effect of mesocarnivore exclusion on cotton rat abundance^50^. We suggest behavioural interactions associated with size-specific spatial segregation mitigate effects on abundance as large rats push small rats into suboptimal habitats where their survival is reduced. Spencer and Cameron^7^ suggested such social interactions may regulate cotton rat populations, especially when density is high. Additionally, our study sites are primarily composed of longleaf pine savannas which are managed with prescribed fire biennially. Cotton rat populations decline sharply after prescribed fires, and mesocarnivore exclusion does not mitigate this decline^50^. Thus, the frequency of prescribed fires on our study site may obscure population-level effects of size-specific survival by effectively re-setting populations every two years. Finally, given that cotton rats are polygynous, larger body size of males, while likely important to the reproductive success of an individual, may not significantly influence population-level dynamics absent stronger effects on female reproductive success.

Body size and growth rates of adult and juvenile female cotton rats did not differ between exclosures and controls. However, there was evidence for size-specific patterns of predation among radio-collared females. Mammalian predators preferentially preyed on large females while raptors preferred small females. Prey choice by predators may be influenced by factors including prey body size^73,95^, activity rates^6,95^, and habitat^73^. Rodent habitat selection may be influenced by body size^7^, age^73^, sex^96^, and breeding condition^96,97^. Female cotton rats in breeding condition select different habitats than males and non-reproductive females to meet their nutritional requirements^96^, and, as described above, dominant cotton rats exclude smaller rats to marginal habitats^7^. A predator’s preference for individual prey characteristics or a preference for hunting in habitat which may be more likely to house individuals of a given sex, size, age, or breeding condition may be difficult to untangle. Dickman *et al*.^73^ observed that barn owls disproportionally preyed on small female house mice (*Mus musculus*) because small females used open habitats more than older mice. Removal of adults led to a reduction in use of open habitats suggesting that mouse social dynamics contributed to the owls’ predation patterns^73^. We suspect a similar combination of social dynamics, habitat selection, and nutritional requirements may explain the sex and size-specific predation patterns seen here, but we lack data to explicitly demonstrate this.

Size-specific predation can profoundly influence population dynamics^59,98^, by influencing growth and maturation^59,99^. However, the size-specific predation patterns we observed in female cotton rats did not influence mean body mass of sampled populations. That we observed an effect of predator exclusion on body mass of males, for which we were unable to detect size-specific predation patterns, while for females we did observe size-specific predation but no effect on body mass, is perplexing. However, we cannot say that size-specific predation did not occur among males as we were only able to radio-collar rats weighing ≥90 g and were therefore unable to monitor predation patterns for a substantial portion of the population. The low prevalence of males weighing 50 to 70 g in exclosures (Fig. 2) suggests size-specific predation likely does influence males which were too small for radio-monitoring.

We investigated how mesocarnivores influenced body mass of cotton rats. Our data suggest adult male cotton rat mass was 9% greater when mesocarnivores were excluded, and this was due to a combination of increased growth rates and increased survival, especially among large males. We suggest this was compounded by social interactions between males. Among female cotton rats, we found no effects on body mass or growth but did find evidence of size-specific predation. Although these responses to mesocarnivore exclusion could conceivably lead to effects at the population level, previous research in these study sites^50^ found little evidence that mesocarnivore exclusion influenced cotton rat abundance, suggesting social interactions and population crashes associated with frequent prescribed fires limited population-level effects. Regardless, our results demonstrate that mesocarnivores influence their prey in multiple ways which can, in combination, have a large effect on body mass.

## Data Availability

All data and code used for analyses in this paper will be archived on the Dryad Digital Repository upon acceptance of the manuscript.
